# Quail FMO3 Gene Cloning, Tissue Expression Profiling, Polymorphism Detection and Association Analysis with Fishy Taint in Eggs

**DOI:** 10.1371/journal.pone.0081416

**Published:** 2013-11-25

**Authors:** Fengtao Mo, Jiangxia Zheng, Peng Wang, Ling Lian, Guoqiang Yi, Guiyun Xu, Ning Yang

**Affiliations:** National Engineering Laboratory for Animal Breeding, MOA Key Laboratory of Animal Genetics and Breeding, College of Animal Science and Technology, China Agricultural University, Beijing, China; Northwestern University Feinberg School of Medicine, United States of America

## Abstract

Quail eggs comprise a significant and favourable part of table eggs in certain countries. Some quail eggs, however, present fishy off-flavor which directly influences their quality. It is reported that flavin-containing monooxygenase 3 (FMO3) is associated with fish-odour trait in human and animal products. FMO3 is responsible for the degradation of trimethylamine (TMA) *in vivo*. Loss-of-function mutations in *FMO3* gene can result in defective TMA N-oxygenation, giving rise to disorder known as “fish-odour syndrome” in human, as well as the fishy off-flavor in cow milk and chicken eggs. In order to reveal the genetic factor of fishy taint in quail eggs, we cloned the cDNA sequence of quail *FMO3* gene, investigated *FMO3* mRNA expression level in various tissues, detected SNPs in the coding region of the gene and conducted association analysis between a mutation and the TMA content in quail egg yolks. The 1888 bp cDNA sequence of quail *FMO3* gene encoding 532 amino acids was obtained and characterized. The phylogenetic analysis revealed quail FMO3 had a closer relationship with chicken FMO3. The *FMO3* mRNA was highly expressed in liver and kidney of quail. Nine SNPs were detected in the coding sequence of quail *FMO3* gene, including a nonsense mutation (Q319X) which was significantly associated with the elevated TMA content in quail egg yolks. Genotype TT at Q319X mutation loci was sensitive to choline. With addition of choline in the feed, the quails with homozygote TT at the Q319X mutation loci laid fish-odour eggs, indicating an interaction between genotype and diet. The results indicated that Q319X mutation was associated with the fishy off-flavor in quail eggs. Identification of the unfavorable allele T of quail *FMO3* gene can be applied in future quail breeding to eliminate fishy off-flavor trait in quail eggs.

## Introduction

Flavin-containing monooxygenases (FMOs, EC 1.14.13.8) are responsible for the oxidation of numerous xenobiotics containing a nucleophilic nitrogen, sulfur, phosphorous, or selenium heteroatom [1,2]. Consequently, FMOs are involved in the metabolic activation or detoxication of numerous xenobiotics including pesticides, drugs, and dietary-derived compounds [3,4]. FMO3 enzyme is considered to be the most important member of the FMO family with respect to the metabolism of foreign chemicals [5] and it is the predominant FMO isoform present in adult human liver involved in the degradation of trimethylamine (TMA) [6]. TMA is thought to be the primary endogenous substrate for FMO3 and a product of the breakdown of dietary chemicals such as trimethylamine *N*-oxide (TMAO), choline, lecithin and betaine by the reductive action of gut bacteria [7]. In human, although other isoforms can oxidize TMA under non-physiologic conditions, TMA is metabolized exclusively to its non-odorous *N*-oxide primarily by FMO3 [8], and is then effectively cleared and excreted in the urine [9]. The individuals with disabled FMO3 cannot oxidize TMA to TMAO in the liver, and that will result in massive amounts of TMA excreted in urine, sweat and breath and present a body odour like the rotting fish, which is called trimethylaminuria (TMAU) or fish-odour syndrome [10,11]. This metabolic disorder disease is inherited in a recessive manner. 

The fish-odour syndrome in human is due to the loss-of-function mutations in *FMO3* gene encoding FMO3 enzyme. Some mutations of the *FMO3* gene can either abolish or diminish the catalytic activity of the enzyme. Since the first P153L mutation in human *FMO3* gene was verified to be associated with TMAU [12], more than 30 distinct causative mutations in human *FMO3* gene have been identified in subsequent studies [13-16]. 

Some animal products such as cow milk and chicken eggs also present fishy off-flavor or fish-odour due to the elevated levels of TMA in them [17,18]. *FMO3* gene had been identified as the causative gene of the fish-odour trait in cow milk and chicken eggs. In cow, a nonsense mutation in exon 6 of *FMO3* gene caused a premature termination of the protein. It seemed that cows with homozygous R238X mutation in *FMO3* ortholog showed a complete lack of FMO3 activity and the TMA cannot be oxidized and accumulated in cow milk, which leaded to the fishy off-flavor in milk of Swedish Red and White dairy breed [19]. In chickens, a nonsynonymous mutation (T329S) was associated with elevated levels of TMA and fishy taint in egg yolks in several chicken lines. It changes an evolutionarily highly conserved amino acid in pentapeptide motif FATGY and may lead to a disfunction of FMO3 enzyme and accumulation of TMA in egg yolks [20]. 

Quail eggs are an important source of nutrients and comprise a significant and favourable part of table eggs in certain countries. The fish-odour occurs occasionally in some quail eggs, and the quail *FMO3* gene is supposed to be the candidate gene for this trait. In this study, we cloned the full-length cDNA sequence of the quail *FMO3* gene and investigated the mRNA expression profile of the quail *FMO3* gene. We also described the variation in the quail *FMO3* gene coding sequence and found a nonsense mutation (Q319X) in exon 7 was highly associated with the elevated TMA content in quail egg yolks, which could be used as an important marker to eliminate the fishy taint in quail eggs in future quail molecular breeding. 

## Results

### Sequence characterization of quail *FMO3* gene

**Figure 1:**
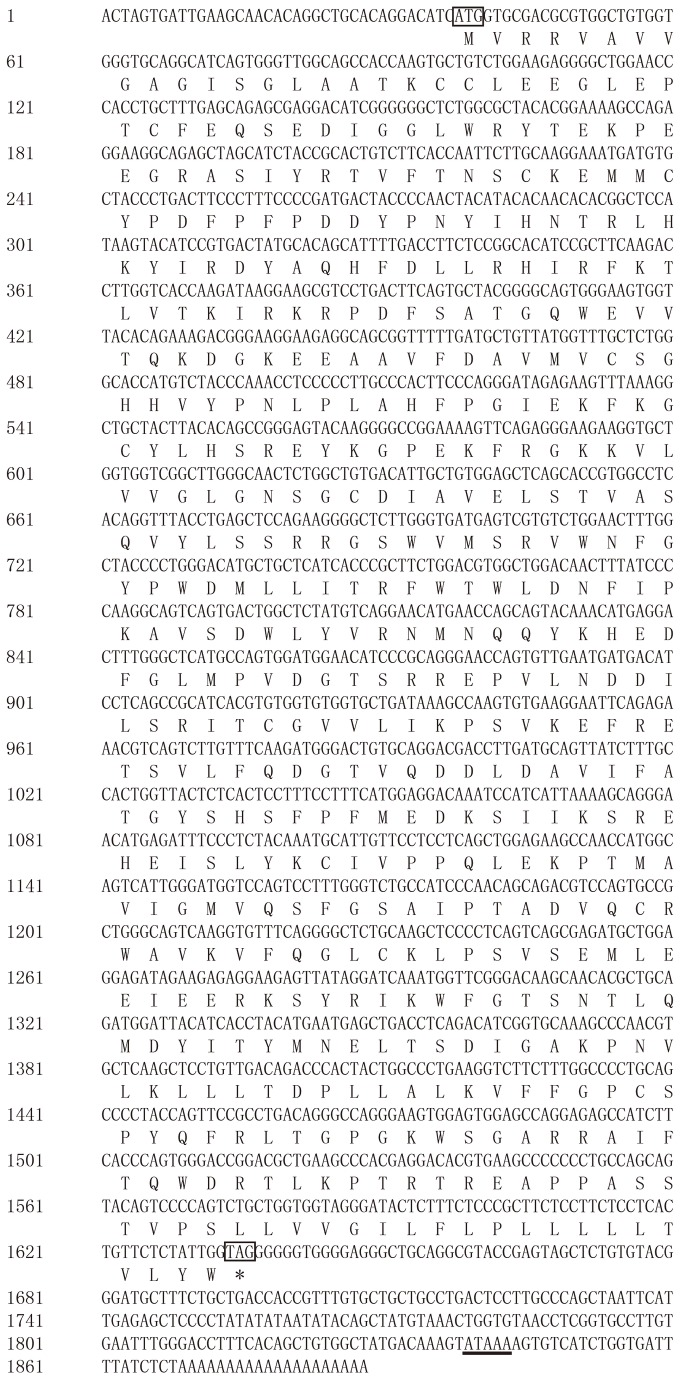
The full-length cDNA sequence of quail *FMO3* gene. The initiator codon and stop codon are showed in black boxes, and the polyadenylation signal sequence is underlined.

After splicing the partial coding region (673 bp), rapid amplification of cDNA ends (RACE) product sequences (3’-RACE and 5’-RACE), a 1888 bp full-length cDNA of quail *FMO3* gene was obtained with a 37bp 5’ untranslated region (5’ UTR) and a 253bp 3’ untranslated region (3’ UTR). It had been deposited into the GenBank database (GenBank: JQ955622). Quail *FMO3* cDNA contained a 1599bp open reading frame (ORF) which encoded 532 amino acid residues (Figure 1). The theoretical isoelectric point (pI) and molecular weight (Mw) of quail FMO3 protein was 8.53 and 60.77 KDa, respectively. The 3’untranslated region included a polyadenylation signal sequence AATAAA (Figure 1). The quail cDNA sequence shared highest identity (92%) to that of the chicken *FMO3* gene (GenBank: NM_204579.1).

**Figure 2:**
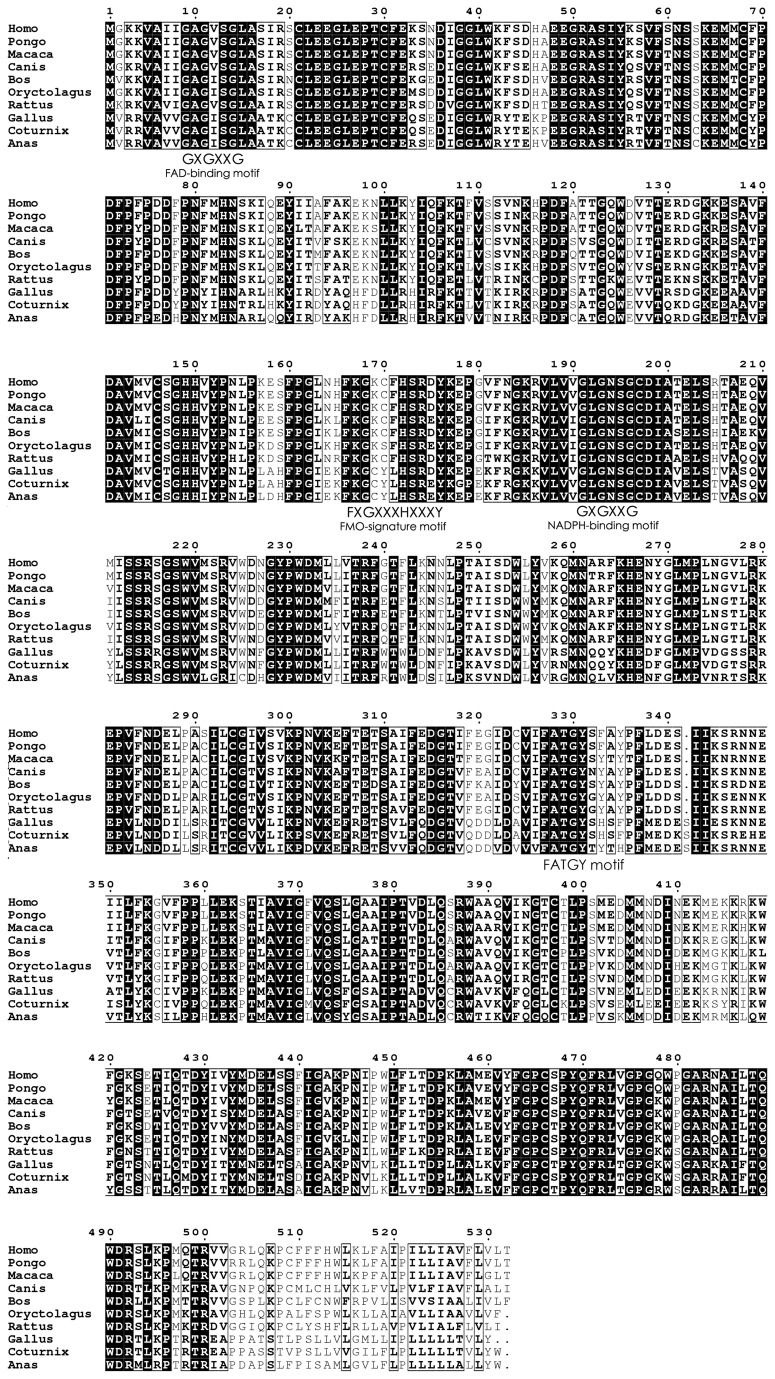
Multiple sequence alignments of FMO3 protein sequences in 10 species. Conserved residues are in black. Boxed residues are semi-conserved. Some important motifs are presented.

The deduced amino acid sequence of quail FMO3 shares the 93% identity with that of *Gallus gallus* (GenBank: NP_989910.1) and 81% with *Anas platyrhynchos* (GenBank: AGC00818.1), and relative low identity with other species, e.g. 63% with that of *Rattus norvegicus* (GenBank: NP_445885.2), 60% of *Mus musculus* (GenBank: NP_032056.1), 61% of *Canis lupus familiaris* (GenBank: NP_001003060.1), 62% of *Oryctolagus cuniculus* (GenBank: NP_001075715.1), 62% of *Macaca mulatta* (GenBank: NP_001028065.1), 63% of *Bos Taurus* (GenBank: NP_776482.1), 61% of *Pan troglodytes* (GenBank: NP_001009092.1), 61% of *Pongo abelii* (GenBank: NP_001124820.1) and 61% of *Homo sapiens* (GenBank: NP_001002294.1). The multiple alignments of duck FMO3 protein with those of other species were presented in Figure 2. The result of multiple sequence aligment revealed quail FMO3 protein sequence includes the FMO-signature motif (FXGXXXHXXXY), FAD-binding motif (GXGXXG), NADPH-binding motif (GXGXXG) and the conserved FATGY motif. These results verified that this cDNA sequence is the cDNA of quail *FMO3* gene. 

### Phylogenetic relationships of FMO3 protein among species

**Figure 3:**
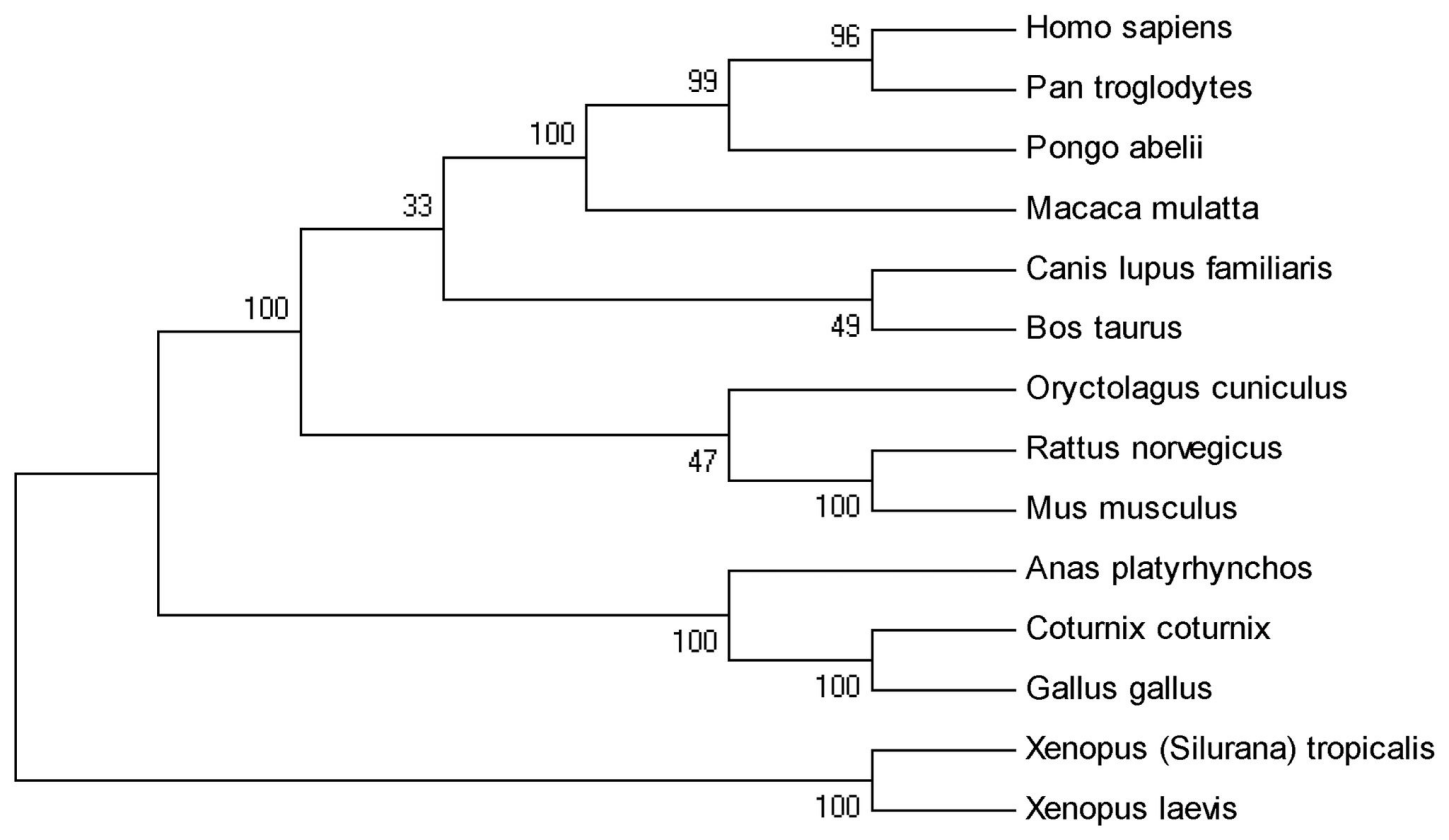
Phylogenetic tree of FMO3 proteins of 14 species. Branches of the phylogenetic tree were labelled with species name.

The phylogenetic tree of FMO3 protein sequences from 14 species could elucidate the genetic relationships of quail FMO3 protein with other species. Based on the Bootstrap Value (Figure 3), all species could be clustered into three different subgroups. While three avian species were grouped into one cluster, quail was closer to chicken than to duck (Figure 3). The result was consistent with previous molecular evolution results, which indicated that chicken can be a good model to study the function of quail *FMO3* gene and protein. 

### *FMO3* mRNA expression profile in quail tissues

**Figure 4:**
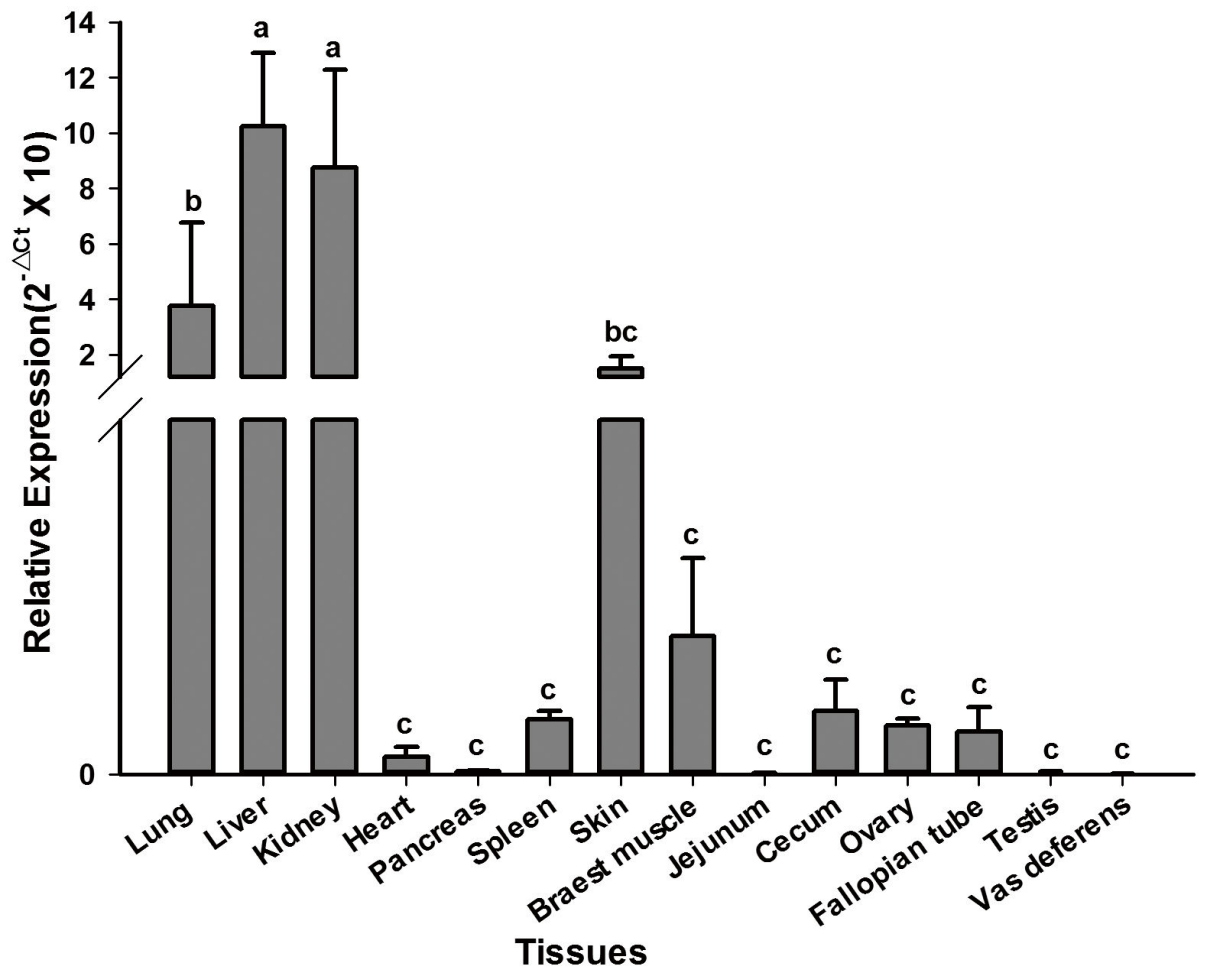
Relative expressions of quail FMO3 mRNA in 14 tissues. Total RNA from 14 tissues of three quails were used to perform the qPCR. qPCR was run in triplicates. The expression of quail FMO3 was normalized to the amount of GAPDH mRNA. Data above were presented as means ± standard deviation (SD). Columns sharing different letters (a, b and c) show significant difference (P < 0.05).

The real-time PCR results showed that quail *FMO3* mRNA was highly expressed in liver and kidney, and intermediately expressed in lung and skin, whereas quite low expression levels were found in the other 10 tissues including heart, spleen, pancreas, jejunum, cecum, fallopian tube, ovary, vas deferens, testis, and breast muscle (Table 1 and Figure 4). The ANOVA analysis results revealed that the mRNA levels of quail *FMO3* in liver and kidney were significantly (*P* <0.05) higher than those in other 12 kinds of tissues (Figure 4). The expression level in lung were significantly (*P* <0.05) higher than heart, spleen, pancreas, jejunum, cecum, fallopian tube, ovary, vas deferens, testis, and breast muscle. 

**Table 1:** mRNA expression data about 14 tissues.

Tissues	Sample Size	Means ± SD (X 10000)*
Lung	3	3750.89±3019.77**^*b*^**
Liver	3	10243.75±2648.48**^*a*^**
Kidney	3	8745.70±3534.96**^*a*^**
Heart	3	9.87±5.28**^*c*^**
Pancreas	3	1.62±0.33**^*c*^**
Spleen	3	30.67±5.04**^*c*^**
Skin	3	1506.69±425.76b^c^
Breast muscle	3	77.71±44.30**^*c*^**
Jejunum	3	0.53±0.25**^*c*^**
Cecum	3	35.60±17.78**^*c*^**
Ovary	3	27.53±3.69**^*c*^**
Fallopian tube	3	23.82±13.63**^*c*^**
Testis	3	0.85±0.62**^*c*^**
Vas deferens	3	0.07±0.01**^*c*^**

* means is the average of three different individuals, and (X 10000) indicates that Means ± SD multiply by 1000.

### SNPs found in quail *FMO3* gene

A total of nine polymorphisms were identified in the coding region of quail *FMO3* gene (Table 2), including one nonsense mutation, one missense mutation, and 7 synonymous mutations. Only the nonsense mutation (SNP 3) and missense mutation (SNP 6) lead to the amino acid change. SNP3 was a single base change (C to T) at position nt992 in exon 7 of cDNA sequence (GenBank: JQ955622), leading to the codon change from CAG to TAG and the corresponding Q (glutamine) to X (stop codon) at position 319 in amino acid sequence (GenBank: AFY98079.1). Thus the nonsense mutation was denoted Q319X.

**Table 2:** Polymorphic sites found in the quail *FMO3* gene by sequencing.

SNP number	Nucleotide positions*	Location	SNPs	Codon change	Amino acid positions*	Amino acid change
1	nt76	Exon 2	C>T	AGG/AGT	13	—
2	nt415	Exon 4	G>A	GAA/GAG	126	—
3	nt992	Exon 7	C>T	CAG/TAG	319	Q→X
4	nt1009	Exon 7	A>G	GCA/GCG	324	—
5	nt1015	Exon 7	C>A	ATC/ATA	326	—
6	nt1023	Exon 7	C>T	ACT/ATT	329	T→I
7	nt1030	Exon 7	C>T	TAC/TAT	331	—
8	nt1096	Exon 7	C>A	CTC/CTA	353	—
9	nt1381	Exon 9	G>A	GTG/GTA	429	—

* nucleotide positions and amino acid positions are reference to the cDNA sequence (GenBank: JQ955622) and the amino acid sequence (GenBank: AFY98079.1), respectively.

The missense mutation (SNP6) changes the codon from T (threonine) at position 329 to I (isoleucine), but we found only three heterozygous individuals for this missense mutation in 140 detected individuals. The mutation frequency of T329I was so low that association analysis couldn’t be carried out. 

### Association of Q319X mutation with the content of TMA in quail eggs

**Figure 5:**
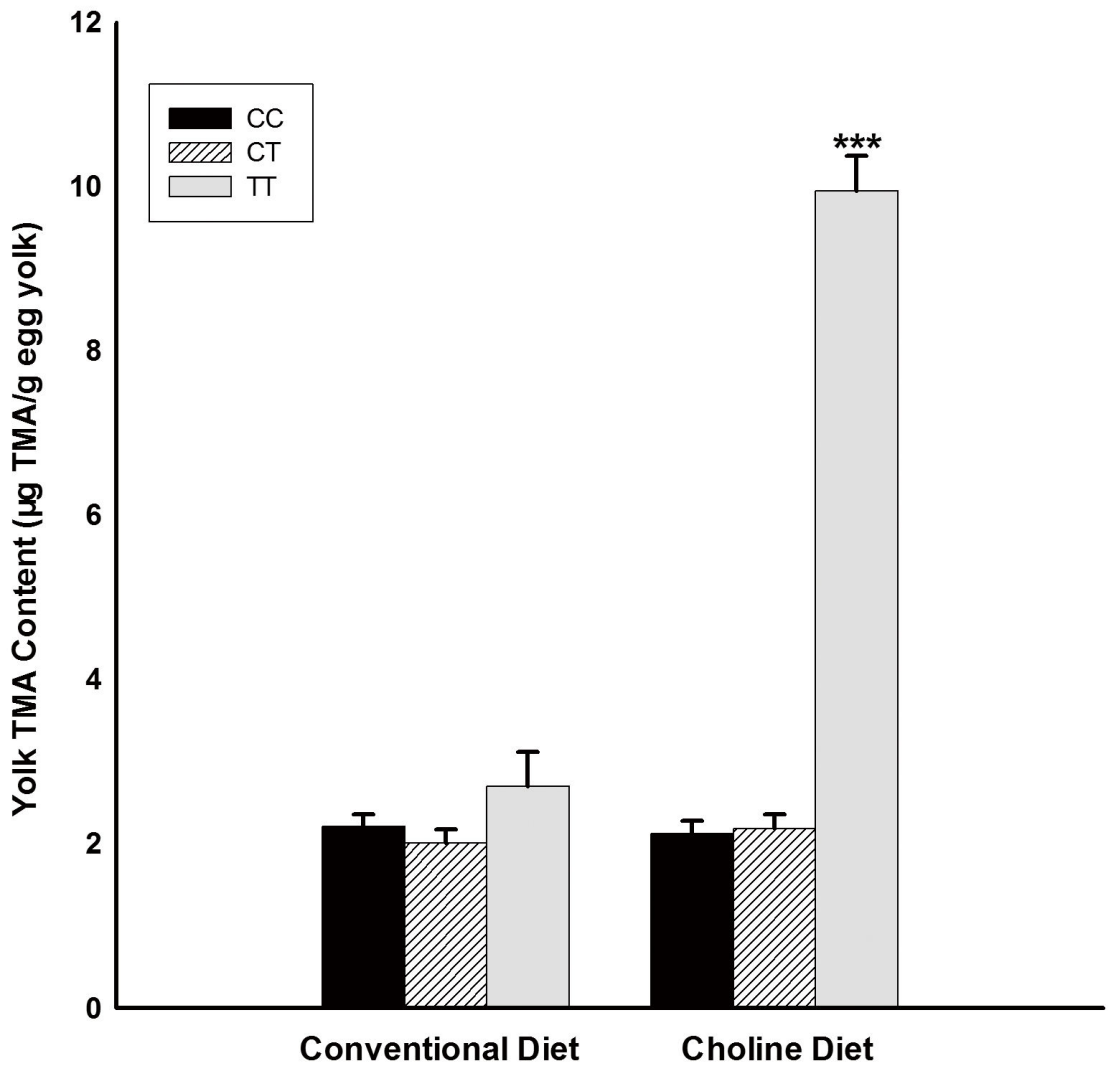
The TMA content of egg yolks of different genotypes in two feed periods. The TMA content of egg yolks are presented as mean ± standard deviation (SD).

The locus Q319X was in Hardy-Weinbery equilibrium (*P* >0.05) in the experimental population of quail (Table 3). Association analysis was conducted to assess whether the genotypes of Q319X mutation in quail *FMO3* gene were associated with the TMA content in egg yolks. Statistical analysis demonstrated that the TMA content in egg yolks of TT genotype on Q319X mutation in the experimental period were significantly higher (*P* <0.001) than in other groups (Figure 5). The TMA contents of egg yolks laid by individuals with TT genotype were more than 9.0 μg/g egg yolks. T allele is the causative mutation for fish-odour in quail eggs. However, there was no significant association between three genotypes with TMA content in quail egg yolk in the adaptation period, and the TMA content in egg yolk was low (< 3.0 μg/g egg yolks), suggesting a significant genotype by diet interaction.

**Table 3:** Genotype and allele frequencies at Q319X of the tested population.

Lines	Number	Genotype frequencies			Allele frequencies		*χ^2^*	*P* value
		CC	CT	TT	C	T		
Dam	81	0.69	0.27	0.04	0.83	0.17	0.2034	0.9033
Sire	59	0.34	0.56	0.10	0.62	0.38	2.0278	0.3628
Combined	140	0.54	0.39	0.07	0.74	0.26	0.0512	0.9747

## Discussion

In the present study, a strategy of reverse transcription PCR (RT-PCR) and 5’-RACE and 3’-RACE method were used to obtain the quail *FMO3* cDNA sequence. The 1888bp cDNA sequence encode a protein of 532 amino acids were obtained from quail liver. The length of quail FMO3 amino acids sequence was same as that of duck, while the chicken FMO3 amino acids sequence lack of the 532^th^ aa compared to quail and duck FMO3 protein sequence (Figure 2). The obtained cDNA sequence was named quail *FMO3* since the deduced quail protein sequence exhibits more than 80% identity with known chicken and duck FMO3 amino acid sequence. The designation of “quail *FMO3*” to name this cDNA is in agreement with the actual nomenclature based on the comparison of the amino acid sequence of the mammalian FMOs [5]. This also suggested that the cDNA sequence we obtained was indeed the quail *FMO3* cDNA sequence. 

The results of multiple sequence alignment of FMO3 amino acid sequence in 10 species showed quail FMO3 putative protein sequence contained some conserved motifs, including FMO-signature motif (FXGXXXHXXXY), FAD-binding motif (GXGXXG), NAD(P)H-binding motif (GXGXXG), and two absolute conserved pentapeptides (EGLEP motif and FATGY motif). All these motifs were crucial to the functional FMO3 protein. The FMO-signature motif, also named FMO-identifying motif and occurred before the NAD(P)H-binding motif (GXGXXG), contributes to NADPH binding and seems to have an additional function [3]. The functions of FAD-binding motif (GXGXXG) and NAD(P)H-binding motif (GXGXXG), two classic Rossmann [21] fold of DBMs, are to bind the ADP moiety and NAD(P)H [22], respectively. The conserved FATGY motif has been recognized for years and has been speculated to be a substrate recognition pocket of FMOs. The T329S change in this motif of chicken *FMO3* gene made the loss-function of chicken FMO3 enzyme [23]. 

The tissue-specific expression profile of quail *FMO3* mRNA was detected by quantitative RT-PCR and the result showed that the quail *FMO3* mRNA was differently expressed in 14 tissues. The expression pattern of quail *FMO3* mRNA was much similar to that in duck, for both the quail and duck *FMO3* mRNA were expressed highly in liver, lung, and kidney [24]. The result was also consistent with results in previous studies, which the *FMO3* mRNA was abundant in adult human liver [25], female mouse liver, rabbit liver, hamster liver and mouse liver, and kidneys of rat, guinea pig and mouse [26]. In human, *FMO3* mRNA was mainly detected in adult liver and the amounts of *FMO3* mRNA in lung and kidney were approximately 4.5%, 3.7% of the amount in adult liver, respectively [27]. While in our study, the amounts of quail *FMO3* mRNA in lung and kidney was 36.8% and 85.8% of that in liver. The tissue-specific steady-state mRNA levels for quail *FMO3* could set the base for future investigations of tissue-specific FMO3 protein levels in quail. 

There are two nonsynonymous SNPs including one missense mutation (SNP 6, T329I) and one nonsense mutation (SNP 3, Q319X) in exon 7, which lead to the change of the corresponding amino acids. The missense mutation (T329I) in quail was found at the same position of the missense mutation (T329S) in chicken FMO3 protein sequence. The T329S substitution of chicken FMO3 protein sequence resided within the evolutionarily highly conserved pentapeptide motif FATGY in mammals was associated with elevated levels of TMA and fishy taint of egg yolks in several chicken lines [28]. The quail T329I substitution was found in the same absolute conserved pentapeptide motif FATGY. But only three heterozygous individuals were detected in140 individuals. Thus we didn’t do the association analysis on this site. 

In quails, the fish-odour is observed only under certain conditions. Feed could affect the trait by providing increased choline, a TMA precursor, which becomes TMA under the metabolism of intestinal microbes. In this study, we found that the genotype of Q319X mutation in *FMO3* gene was highly associated with the TMA content in egg yolks. The Q319X mutation causes a premature termination, eliminating about 40% of the quail FMO3 enzyme which contains 532 amino acids, and will lead to the loss of function of the enzyme. Similar situation had been found on dairy cattle [19], the nonsense mutation R238X in *FMO3* gene also made a premature termination and caused the fishy off-flavor in milk. In human, two nonsense mutations (E305X, E314X) had been verified lead to a complete loss of enzyme activity and lead to the TMAU in human [15]. When the quails were feed with high level of choline, the TMA content in quail eggs laid by individuals with TT genotype was much more than those with CC or CT genotypes. All the eggs laid by individuals with TT genotype had elevated TMA content (more than 9.0 μg/g egg yolk) and presented the fish-odour like chicken eggs. By the molecular screening procedure established in this study, the unfavourable allele T can be identified and eliminated completely in the breeding stocks of quails, which will lays eggs without fish-odour even fed the feed with the sufficient TMA precursors like choline.

## Materials and Methods

### Ethics statement

Animal experiments were approved by the Animal Care and Use Committee of China Agricultural University and the experiment was performed according to regulations and guidelines established by this committee.

### Sample Collection

Three male and three female Japanese quails (20 weeks of age), provided by Hubei Shendan Healthy Food Co., Ltd, were used for *FMO3* gene cloning and mRNA expression analysis. All these quails were slaughtered at the same time, and 14 different tissues, including heart, liver, spleen, lung, kidney, pancreas, jejunum, cecum, fallopian tube, ovary, vas deferens, testis, breast muscle, and skin were collected and kept in liquid nitrogen immediately, and then stored at -80 °C for total RNA extraction.

For SNP detection and association study, female quails from a sire line and a dam line of a Japanese quail crossbreeding package were kept in single cages for laying records. A feeding experiment was performed on 81 female quails (37 weeks of age) from the sire line and 59 female quails (37 weeks of age) from the dam line. A total of 15 days of feeding experiment was divided into two stages, an adaptation period (first five days) and an experimental period (next 10 days). A conventional diet was formulated according to the feeding standard of laying quail. In the experimental period, additional choline chloride (4000 mg/kg) was added into the diet. During adaptation period and the last five days of the experimental period, at least three yolks from each quail were collected and stored at 4 °C for TMA analysis. Blood of each quail was collected and stored at -20 °C for DNA extraction. 

### RNA isolation and cDNA synthesis

Total RNA was extracted from a liver tissue by using TRIZOL total RNA extraction reagent (Invitrogen, US) according to the manufacturer’s protocol. The quality of the RNA was assessed by 1% agarose gel electrophoresis using 1 µl of RNA. The concentration was detected by Nanovue (GE Healthcare, US). 

The first strand cDNA was synthesized from purified RNA by reverse transcription PCR (RT-PCR). One microgram of total RNA was reversely transcribed by M-MLV reverse transcriptase (Promega, Madison, WI), oligodT primer (10 pmol/µl) and Ribonuclease Inhibitor (RRI) in a total volume of 50 µl reaction according to the manufacturer’s instructions. The reaction conditions included 25 °C for 10 min, 37 °C for 60 min, 95 °C for 5 min, and 20 °C for 5 min. 

### Cloning of cDNA of quail *FMO3* gene

Based on the most conserved regions of the sequences in different species, a pair of primers was designed to amplify the partial coding region of quail *FMO3* gene. PCR amplifications were conducted in a final volume of 15 µl with 1.2 µl first strand cDNA, 7.5 µl 2×Taq PCR Master Mix and 0.2 µl of each primer (10 pmol).The optimum PCR amplification conditions were programmed as5 min pre-denaturation at 94 °C, followed by 35 cycles of denaturation at 94 °C for 30 sec, annealing at 63 °C for 30 sec, extension at 72 °C for 60 sec, then final extension at 72 °C for 8 min. The PCR products were subjected electrophoresis on 1% agarose gel. The principal product (673 bp) was gel-purified, subcloned into pMD-19T vector (TaKaRa, Dalian, China), and sequenced by GENEWIZ Co. Ltd. (Beijing, China).

Subsequently, 3’-RACE and 5’-RACE were performed to obtain the full-length of quail *FMO3* mRNA sequence. The SMART 5’-RACE and 3’-RACE cDNA amplification kit (Clontech, USA) was used according to the manufacturer's recommendations. Two gene-specific primers (GSP1 and GSP2, Table 4) used were designed based on the sequence obtained, then the 3’ end cDNA sequence and 5’ end cDNA sequence were synthesized by nested PCR, respectively. The PCR reaction was conducted in a total volume of 50 µl and the following touchdown PCR amplification was performed at follows: firstly with 5 cycles of 94 °C for 30 sec and 72 °C for 30 min, then 5 cycles of pre-denaturation at 94 °C for 3 sec, annealing at 68 °C for 30 sec, and extension at 72 °C for 3 min, finally with 30 cycles of 94 °C for 30 sec, 67 °C for 30 sec, 72 °C for 3 min. The RACE PCR products were gel-purified, subcloned into pMD-19T vector (TaKaRa, Dalian, China), and sequenced by GENEWIZ Co. Ltd. (Beijing, China). At least five independent clones were sequenced for each PCR product to ensure the accuracy of sequencing. After got the full length of *FMO3* gene by aligning and splicing, a pair of primers for the full cording sequence was designed to verify the correctness of the obtained full length cDNA sequence of *FMO3* gene. 

**Table 4:** Primers used for cloning and real-time PCR.

Name	Sequence (5’→3’ )	Function
FMO3-p	F: GCCGGAAAAGTTCAGAGG	Partial coding region
	R: TGAGGGGAGCTTGCAGAG	
GSP1	CCGCTGGGCAGTCAAGGTGTTTC	3’ RACE
GSP2	CCACCAGCACCTTCTTCCCTCTG	5’ RACE
FMO3-f	F: GAAGCAACACAGGCTGCACAGGAC	Full coding region
	R: CAGCAGAAAGCATCCCGTACACAG	
FMO3-q-PCR	F: ATAGGATCAAATGGTTCGGGACAA	q-PCR
	R: ACCTTCAGGGCCAGTAGTG	
GAPDH-q-PCR	F: GGGTGGTGCTAAGCGTGTTATC	q-PCR
	R: GACCCTCCACAATGCCAAAG	

### Sequence analysis

According to the three sequenced fragments, the full-length cDNA sequence was assembled using DNAMAN software. Open reading frame (ORF) and amino acid sequences were deduced using ORF Finder (http://www.ncbi.nlm.nih.gov/gorf/). The theoretical pI and Mw of protein was computed using Compute pI/Mw Tool (http://web.expasy.org/compute_pi/). Nucleotides as well as the derived amino acid sequences of duck *FMO3* gene were blasted with those reported *FMO3* sequences of different species at National Center for Biotechnology Information (NCBI) server (http://www.ncbi.nlm.nih.gov/BLAST). Multiple sequence aligment was analyzed by Clustal X. 

### Phylogenetic analysis

Using neighbor-joining (NJ) method with Molecular Evolution Genetics Analyses (MEGA) version 5.0, a phylogenetic tree was constructed based on the multi-sequence alignments of the FMO3 protein sequences of 14 species including quail (*Coturnix coturnix*), chicken (*Gallus gallus*), duck (*Anas platyrhynchos*), human (*Homo sapiens*), chimpanzee (*Pan troglodytes*), orangutan (*Pongo abelii*), macaque (*Macaca mulatta*), dog (*Canis lupus familliaris*), cow (*Bos taurus*), rabbit (*Oryctolagus cuniculus*), rat (*Rattus norvegicus*), mouse (*Mus musculus*) and two kinds of Xenopus (*Xenopus tropicalis* and *Xenopus laevis*).

### *FMO3* gene expression analysis in distinct quail tissues

Total mRNA from 14 different tissues of three quails, including heart, liver, spleen, lung, kidney, pancreas, jejunum, cecum, fallopian tube, ovary, vas deferens, testis, breast muscle, and skin, were extracted to investigate the mRNA expression profile of the quail *FMO3* gene using real-time PCR. Each qPCR reaction was run in triplicates. Quail *GAPHD* gene was used as the internal reference gene. The primers for *FMO3* gene and *GAPDH* gene were designed by Primer 5.0 software (Table 4). 

Real-time PCR was performed on the ABI 7300 system (Applied Biosystems, USA) using Power SYBR® PCR Master Mix (Applied Biosystems, USA). All real-time PCR reactions were run in triplicate. The PCR reactions were performed in a final volume of 15 µl with 1.2 µl of cDNA, 0.2 µl of each gene-specific primer, 7.5 µl 1x PCR mix and 5.9 µl purified water. The optimum thermal cycling procedure was 50 °C for 2 min, 95 °C for 10 min, 40 cycles of 95 °C for 15 sec, 60 °C for 1 min, 95 °C for 15 sec, 60 °C for 30 sec, and 95 °C for 15 sec. The relative expression of duck *FMO3* mRNA was calculated relative to the amount of *GAPDH* present. 

The real-time PCR data were analyzed using comparative Ct method [29]. The Ct values were means of triplicate samples tested. The gene expression was calculated as 2^-∆Ct^ (∆Ct =Ct target – Ct internal control) and the value indicated an n-fold difference relative to the expression of the internal control gene. Differential expressions among 14 tissues were analyzed by ANOVA program of SAS 9.2 version. Multiple comparison analysis was conducted using Duncan method. Comparisons were considered significant at P < 0.05.

### SNP detection of quail *FMO3* gene by sequencing

In total, 140 quails were used to detect mutations in quail *FMO3* gene. The primers (Table 5) covering the coding region of quail *FMO3* gene were designed based on the obtained quail *FMO3* cDNA sequence. The PCR were performed in 20 µl of reaction volume with DNA, Taq DNA polymerase, PCR buffer with MgCl_2_ and primers. Then the reaction was incubated at 94°C for 5 min, 35 cycles of 94°C for 30 sec, 62-65 °C (Table 5) for 30 sec, and 72°C for 45 sec, followed by a final extension at 72°C for 10 min. After sequencing, the sequences were aligned to identity the polymorphisms in quail *FMO3* gene.

**Table 5:** Primers used for SNPs detection.

Primers	Sequences (5’→3’)	Tm (°C)	Length (bp)
Primer1	F: ATGGTGCGACGCGTGGCTGT	64.1	137
	R: TTCCGTGTAGCGCCAGAGCC		
Primer2	F: CAGAGGAAGGCAGAGCTAGC	64.1	645
	R: GTAAAGTGCACACCTGGCTG		
Primer3	F: GGGATAGAGAAGTTTAAAGGTTG	65.4	267
	R: ACACGGCTCATCACCCAGGA		
Primer4	F: TCCTGGGTGATGAGCCGTGT	64.1	386
	R: GATGTTTCTCTGAATTCCTTCAC		
Primer5	F: GTGAAGGAATTCAGAGAAACG	62.0	279
	R: CTGAAACACCTTGACTGCC		
Primer6	F: TGGTTCGGGACAAGCAACAC	65.0	337
	R: TAGAGCACAGTGAGGAGGAG		

### TMA content in egg yolks

The procedures for determination of TMA concentration in egg yolk followed that in chickens [20]. Briefly, TMA was extracted from egg yolks using 10% trichloroacetic acid (TCA, 1ml/g egg yolk), and 2 ml of extract was made alkaline by adding 1.5 ml 50% potassium hydroxide to separate the amine from its salts. To fix other nitrogen-containing components, 0.5 ml 10% formaldehyde was added. The free TMA was dissolved in 5 ml of toluene. A 2.5 ml aliquot of the supernatant was mixed with an equal volume of 0.02% picric acid to form a yellow TMA-N-picrate complex. It was subsequently measured at 410 nm using ultraviolet spectrophotometer. A standard curve (R^2^=0.9899) was produced from 14 TMA standard solutions (ranging from 0 to 20 µg/ml of TMA-N) to calculate the TMA-N concentration of egg yolks. The TMA content was then presented as TMA-N in egg yolks.

### Genotyping and association analysis

The SNPs were genotyped by PCR and directly sequenced. The PCR reaction procedure was same as the program depicted in SNPs detection. The association between genotypes of SNPs in *FMO3* gene and TMA content in egg yolks were calculated with the general linear model (GLM) procedure of SAS 9.2 version. The model: *Y*_*ijk*_=µ + *G*_*i*_ + *L*_*j*_ + *e*_*ijk*,_ where *Y*_*ijk*_ is the observation of the TMA content of egg yolks, µ is the least square means, *G*_*i*_ is the effect of *i*th genotype (=1-3), *L*_*j*_ is the effect of *j*th line (sire line and dam line) and *e*_*ijk*_ is the effect of the random residual. Then multiple comparison analysis was conducted to compare TMA content in quail eggs of each genotype by using Least Squares Means. 

## Conclusions

In this study, the full-length cDNA sequence of quail *FMO3* gene was successfully cloned and both the nucleotide sequence and the putative amid acid sequence were analyzed. The quail *FMO3* mRNA expression profile in 14 tissues was also detected. Nine SNPs were identified in different exons and there was a highly significant association between the TMA content of quail egg yolks and Q319X mutation, which led to the fish-odour of quail egg yolks. Here, we found an important marker and present an effective method to identify potential “fish-odour” individuals by direct testing of *FMO3* genotype irrespective of the age, environmental factors, or gender of the tested individuals, which will help in eradicating the genetic factor behind fish-odour in quail eggs and lay a good foundation for further study and the molecular breeding for quail. 
